# Freshwater and Sediment Host Distinct Yet Overlapping Microeukaryotic Communities, With Sediment Communities Less Impacted by Treated Wastewater

**DOI:** 10.1111/jeu.70070

**Published:** 2026-02-24

**Authors:** Radtke Kim, Bludau Dana, Boenigk Jens, Sieber Guido

**Affiliations:** ^1^ Department of Biodiversity University of Duisburg‐Essen Essen Germany; ^2^ Centre for Water and Environmental Research University of Duisburg‐Essen Essen Germany; ^3^ Department of Engineering and Natural Sciences Westphalian University of Applied Science Recklinghausen Germany

## Abstract

Freshwater and sediment environments host diverse microeukaryotic communities that differ in structure and composition, yet exhibit taxonomic overlap. Using 18S V9 rRNA gene sequencing, we compared communities from these habitats in controlled 10‐day mesocosm experiments, examining diversity, overlap, and responses to treated wastewater (TWW). Habitat type was the strongest determinant of community composition: sediments displayed higher diversity and greater temporal stability than freshwater communities. While many taxa were shared, highly dominant OTUs were mostly rather habitat‐specific, whereas taxa occurring evenly across both habitats were generally rare. Distinct trophic structures further distinguished the habitats, with sediments showing relatively balanced assemblages of phototrophs, mixotrophs, consumers, and parasites, while freshwater communities were dominated by consumers. TWW exposure induced pronounced but transient changes in freshwater communities, including an initial increase in richness from allochthonous taxa, followed by partial convergence toward controls, whereas sediment communities remained largely unaffected. We identified 14 taxa associated with TWW, nine of which have not previously been linked to wastewater, highlighting their potential as bioindicators. Our findings reveal contrasting sensitivity and resilience of freshwater microeukaryotic habitats and emphasize the importance of integrating both water column and sediment communities in monitoring and assessing the ecological impacts of treated wastewater.

## 1 Introduction

Microeukaryotes are ubiquitous organisms that inhabit virtually every environment on Earth—from soils and sediments to freshwater, marine, and even extreme habitats such as hot springs (Da Carvalho Silva and Fernandes 2023; Grossmann et al. 2016; Rappaport and Oliverio 2023). Despite their small size, they play a major role in ecosystem functioning by driving primary production, nutrient cycling, and energy flow. Their activities support ecosystem health and stability, making them key players in the maintenance of biodiversity and the provision of essential ecological services that ultimately sustain human societies (Geisen et al. 2018; Mitra et al. 2016).

Given the central role microeukaryotes play in ecosystem processes, understanding their dynamics in critical habitats such as freshwater systems is essential. Freshwater is essential for human needs such as drinking, agriculture, and sanitation, yet it is a limited resource that accounts for only about 2.5% of the Earth's water (Gleick 1993). It is affected by natural factors—such as flow, turbidity, and light availability—as well as anthropogenic stressors like agricultural runoff and treated wastewater, placing this vital resource increasingly at risk. Sediments, as an integral part of freshwater ecosystems, are also exposed to both natural variability and human‐induced pressures (Beattie et al. 2020; Świacka et al. 2023; Zhang et al. 2023).

Although sediments and the overlying water column are physically connected, they differ substantially in structure and ecological function. Sediments, due to their structural similarity to soils, offer greater habitat complexity, more stable environmental conditions, and increased resource availability than the water column (Geisen et al. 2018; Wu et al. 2023). These properties are known to promote higher microbial diversity, as has been demonstrated in marine environments (Fang et al. 2021; Schoenle et al. 2021). At the same time, freshwater and sediment habitats are functionally interconnected: sediments can act as reservoirs for nutrients, while the overlying water supplies oxygen and energy‐rich compounds to deeper sediment layers (Beattie et al. 2020; Cardoso et al. 2019; Forsberg 1989; Higashino et al. 2008). Consequently, disturbances affecting one of these habitats may directly or indirectly impact the other—or have no effect at all (Boden et al. 2025, 2023; Graupner et al. 2017; Simon et al. 2016). However, although these habitats are closely linked, the effects of stressors across both habitats remain insufficiently studied, as most research tends to focus on only one compartment.

Despite the clear ecological distinction between sediment and water, overlaps in community composition can occur due to close geographical proximity, drift, or other factors. While such overlaps have been studied in soil–freshwater systems (Sieber et al. 2020; Singer et al. 2021), the extent to which sediment and water communities share taxa is still poorly understood. Since sediment dynamics influence the quality and stability of neighboring aquatic habitats, understanding the interaction and potential overlaps between species that occur in sediments and those that live in the water column is essential for comprehending overall population dynamics (Che et al. 2024; Fang et al. 2021; Shi et al. 2020). Given their close and continuous contact, aquatic sediments may share a higher number of taxa with freshwater habitats compared to soil and may even serve as microbial seed banks following disturbance events, potentially supporting recovery processes in both habitats (Shi et al. 2020; Simon et al. 2016; Wu et al. 2023). Understanding these dynamics is essential for interpreting stressor responses in coupled aquatic systems.

Nevertheless, despite this overlap and the potential for inoculation between the two habitats, we assume that they remain clearly distinguishable based on their overall community composition, which may reflect the presence of different organisms or varying abundances of shared species (Burki et al. 2021). However, the functional characteristics related to nutritional modes of the organisms in water and sediment environments also remain largely unknown. This is particularly relevant in the context of environmental stress, as different trophic strategies may be associated with varying degrees of sensitivity or resilience (Boden et al. 2024; Sieber et al. 2022).

One major threat that introduces a cascade of effects is treated wastewater, which is associated with pollution, eutrophication, and the introduction of microorganisms that are either absent or much less abundant in pristine environments (Etsuyankpa et al. 2024; Koul et al. 2022; Sieber et al. 2023; Stach et al. 2023). With increasing global population and urbanization, wastewater production is expected to rise further (Bonetta et al. 2022). In addition, a substantial number of rivers and streams already consist of high proportions of treated wastewater (Guillet et al. 2019). As freshwater scarcity intensifies, the reuse of treated wastewater will become essential to meet human water demands, making its reintroduction into natural freshwater systems inevitable (Christou et al. 2024). Understanding the ecological consequences of this development is therefore critical for designing reliable monitoring tools and mitigation strategies. While treated wastewater is already known to cause pronounced shifts in microbial community composition in the water column (Guillet et al. 2019; Shah et al. 2025; Sieber et al. 2023; Stach et al. 2023), its impact on sediment‐associated communities remains largely unexplored.

We assume that the sediment will be less impacted, as primarily water—not sediment or sludge—is discharged from wastewater treatment plants and sediments are known to withstand and recover from anthropogenic stressors (Boeraș et al. 2024; Clark et al. 2025). However, since the water column inevitably interacts with the sediment, effects may also become apparent there, albeit to a lesser extent.

The introduction of treated wastewater is expected initially to increase species richness in freshwater habitats, as novel microbial taxa—often absent in pristine environments—are introduced. Over time, richness may decline as communities adjust, and non‐adapted species are outcompeted or die off (Chouari et al. 2017; Gad et al. 2024; Payne 2013). Particularly, heterotrophic microeukaryotes are predicted to increase in abundance, as they play a key role in nitrogen removal processes within wastewater treatment systems and are commonly associated with these environments (Foissner 2016; Pérez‐Uz et al. 2010; Rozo‐Montoya et al. 2023).

To test our hypotheses, six mesocosm systems simulating a stream with freshwater and sediment were constructed. Treated wastewater was added to half of the mesocosms to examine how microeukaryotic communities in both habitats respond to the addition, and to investigate the extent of organismic overlap between the water column and sediment. Further, we aimed to identify indicative species which could be used for environmental monitoring to detect treated wastewater in receiving waters, as microeukaryotes hold great potential as bioindicators in aquatic systems (El‐Tohamy et al. 2024; Foissner 2016; Mostafa et al. 2023; Payne 2013).

## 2 Methods and Materials

### 2.1 Setup

The raw sequencing data used for the analysis of microeukaryotic communities in freshwater and sediment habitats originate from the AquaFlow treated wastewater experiment described in detail by Sieber et al. and Bludau et al. (Bludau et al. 2025; Sieber et al. 2023). The publicly available sequencing data were retrieved from the NCBI Sequence Read Archive (SRA; accession number PRJNA1019091).

Briefly, the study was conducted in six identical circular‐flow mesocosms (“AquaFlow systems”) located in the greenhouses of the University of Duisburg‐Essen. Each system contained 365 L of prefiltered river water (200 μm) and 60 L of homogenized river sediment from the Boye River (Germany, 51°33′19.7″N 6°56′38.3″ E). Each circular‐flow system consisted of three interconnected water tanks (40, 40, and 270 L) linked by two sediment‐filled channels measuring 10 cm in width and 4 and 2 m in length, respectively. After a 14‐day acclimatization period with daily interconnection of all systems to homogenize conditions, three mesocosms (T1, T2, T3) were treated with 33% treated wastewater (TWW) from a local wastewater treatment plant, while the remaining three served as controls (C1, C2, C3). All systems ran in parallel under controlled conditions (19°C, natural light) for 10 days. This setup allows controlled simulation of TWW impact on aquatic microbial communities over time.

### 2.2 Sampling

Each AquaFlow system was sampled at seven time points. Water samples (Sieber et al. 2023) were collected after 1 h, 12 h, 1 day, 2 days, 4 days, 7 days, and 10 days (S1–S7), while sediment samples (Bludau et al. 2025) were taken after 1 h, 4 days, and 10 days. In detail, 400 mL of water was filtered using a 0.2‐μm polycarbonate filter. Water was taken from the 270‐L tank and not directly above the sediment to minimize the risk of sediment reaching the filter due to potential resuspension. For the same reason, water samples were always collected before sediment sampling. Sediment was sampled by removing three cores per mesocosm and per sampling point using stainless‐steel mesh cylinders (9‐cm height, 3‐cm diameter, 2‐mm mesh size) that had been initially inserted into the sediment. The sediment from these three cores was pooled to create a representative sample. Prior to core removal, slightly larger steel tubes were inserted into the sediment to delineate the sampling area and prevent sediment from collapsing. After removal of the three mesh cores, the extracted sediment was replaced with autoclaved sand.

For comparative analyses, only time points sampled in both habitats (S1 = 1 h, S5 = 4 days, S7 = 10 days) were used. For water, 400 mL were filtered through 0.2‐μm polycarbonate filters (Cytiva, Nucleopore), air‐dried, preserved in 400‐μL DNA/RNA Shield (Zymo Research), flash‐frozen in liquid nitrogen, and stored at −80°C. Integrated sediment samples (10 cm depth) were taken in triplicate, homogenized, and frozen prior to DNA extraction.

### 2.3 DNA Extraction and Amplification

DNA from water associated organisms was extracted using the Zymo Quick‐DNA/RNA Microprep Plus Kit with a modified protocol (Sieber et al. 2022), while DNA from sediment‐associated organisms was extracted as described in Bludau et al. (2025). For the sediment and water samples, a PCR amplification was conducted to amplify the 18S V9 rRNA region using forward primers based on 1391f (5′‐GTACACACCGCCCGTC‐3′) (Amaral‐Zettler et al. 2009) and the reverse primers which were based on. EukR (5′‐TGATCCTTCYGCAGGTTCACCTAC‐3′) (Medlin et al. 1988). The PCRs were performed using the QIAGEN Multiplex PCR Kit (Qiagen, Germany) with 12.5 μL 2× Qiagen Multiplex PCR Master Mix, 2.5 L primer, or 1.25 L primer each (0.2 μL), 1 μL DNA template, 5 μL 5× Q‐Solution and 4 μL RNAse‐free water. For the detailed PCR protocol, please refer to Sieber et al. (2023) and Bludau et al. (2025).

### 2.4 Sequencing

Water samples were sequenced on an Illumina NovaSeq6000 and sediment samples on an Illumina NextSeq 500, both yielding 150‐bp pair‐end reads (Fasteris, Geneva, CH) (Bludau et al. 2025; Sieber et al. 2023). Adapter removal, demultiplexing via MID sequences, and initial quality trimming were performed by the sequencing company. Sequence quality was assessed using FastQC (Andrews 2010).

Raw reads were processed with the Natrix2 Pipeline (Deep et al. 2023) to cluster them into operational taxonomic units (OTUs). Reads were quality‐filtered using a minimum Phred score of 25, a minimum read length of 100 bp and a maximum length of 300 bp. Paired‐end reads were then assembled using PANDAseq (Masella et al. 2012) using a minimum read overlap of 15 bp, an assembly threshold of 0.9 and a minimum quality score of 1 for bases within the assembled reads. Sequences only present in both technical PCR replicates after dereplication were retained to minimize sequencing errors. OTUs were clustered using SWARM with default parameters (Mahé et al. 2014). All sequences were taxonomically assigned to the PR^2^ database Version 5.0.0 (Guillou et al. 2013) using mothur (Schloss et al. 2009). If not stated otherwise, default parameters of the mentioned tools were applied.

### 2.5 Filtering

To reduce noise, OTUs only present in one sample and those with a joint abundance across all samples of less than 100 reads were filtered out. OTUs belonging to plastid DNA, mitochondrial DNA, nucleomorph DNA, chromatophore DNA, as well as OTUs from Bacteria, Embryophyta, Archaea and metazoans were removed. For verification of a sufficient sequencing depth, rarefaction curves were plotted for both water and sediment samples (Figure S2). In addition, a bootstrap confidence threshold of ≥ 60, as assigned by mothur, was used to retain reliable taxonomic assignments (Bludau et al. 2025).

### 2.6 Data Analysis and Visualization

All statistical analyses were conducted in R (R Core Team 2022). Raw abundance data were used to calculate α‐diversity. We used a Shapiro–Wilk normality test (R Core Team 2022) which revealed that the α‐diversity values were not normally distributed. Therefore, non‐parametric tests were used. To test for differences in α‐diversity between water and sediment, as well as between control and treatment groups, we conducted pairwise Wilcoxon tests (R Core Team 2022) and corrected for multiple comparisons using the Holm method. The Shannon–Wiener‐Index (*E* = *H*′/ln[*S*]) was calculated for each sample and then used to calculate species richness (Oksanen et al. 2025) and the effective number of species (ENS) (Oksanen et al. 2025) to test for differences between water and sediment as well as control and treatment samples. Indicative species for treated wastewater, water and sediment were analyzed using the R IndicSpecies package (de Cáceres 2009).

Bray–Curtis dissimilarity (Oksanen et al. 2025) was used to quantify β‐diversity between samples (Kew et al. 2022; Riedel et al. 2016). For β‐diversity analyses only, count data were normalized using DESeq2 (design = ~habitat + treatment) (Love et al. 2014), followed by log‐transformation using the natural logarithm. Principal coordinate analysis (PCoA) (McMurdie and Holmes 2013) was performed to visualize the Bray–Curtis dissimilarity between the habitats, treatments and timepoints. Differences in microbial community composition were assessed using permutational multivariate analysis of variance (PERMANOVA) based on Bray–Curtis dissimilarities. The main effects and interaction between habitat and treatment were tested, as well as treatment effects within individual habitats. To account for temporal variation, all permutation tests were stratified by timepoint with 999 permutations. PERMANOVA analyses were performed in R using the adonis2 function from the “vegan” package (Oksanen et al. 2025). For the differential abundance analysis with DESeq2, *p*‐values were adjusted using the Benjamini–Hochberg (BH) procedure, and OTUs were considered significant if they had a minimum log_2_ fold change of 2 and an adjusted *p* ≤ 0.05.

Whether an OTU was considered shared between habitats or treatments was determined based on its presence or absence in both groups. To assess whether an OTU was more strongly associated with one habitat or treatment, read counts per sample were rarefied (using the rarefy function from the “vegan” package) to the median read abundance across all samples (1,860,826) to account for differences in sequencing depth. An OTU was then considered predominantly associated with a specific group if more than 50% of its total reads were found in that group. The nutrition modes of individual groups and organisms were inferred from Sieber et al. (Sieber et al. 2022; Singer et al. 2021).

The final data visualization was done in R (R Core Team 2022) using the packages ggplot2 (Wickham 2016), tidyverse (Wickham et al. 2019), ggvenn (Yan 2021), phyloseq (McMurdie and Holmes 2013), ggforce (Pedersen 2023), ggpubr (Kassambara 2023). Furthermore, we used CoralDRAW for visualization (Version 25.2.1.313). The authors utilized ChatGPT to improve the readability and grammar of this work during its preparation. Following its use, the authors carefully reviewed and revised the content as necessary and take full responsibility for the final version of the publication.

## 3 Results

### 3.1 Sequence Count

After filtering, a total of 66,989,700 assembled reads remained, clustered into 4169 OTUs. Of these, 2969 OTUs were detected in water samples, and 3772 OTUs were found in sediment samples.

### 3.2 α‐Diversity

Comparison of α‐diversity based on OTU richness revealed that sediment samples generally exhibited higher OTU richness than water samples (*p* ≤ 0.05) (Figure S3). The only exception was at sampling timepoint one in the “water treatment” samples, which showed no significant difference compared to sediment samples (*p* > 0.05). A decline in OTU richness over time was apparent for all groups.

Generally, no significant differences in OTU richness were detected between water samples with and without TWW, nor between sediment samples with and without TWW (*p* > 0.05). Analysis of the individual time points revealed a single significant difference between control and treatment water samples at the first sampling point (*p* ≤ 0.05), while this was not the case for sediment (*p* > 0.05). Additionally, a loss of OTU richness over time was observed in both water and sediment samples; however, it was more pronounced in the water samples (Figure S3).

Between control and treatment samples within each habitat, the effective number of species (ENS) remained relatively similar (Figure S4), reflecting the patterns observed in OTU richness. In detail no significant differences between the treatment and control group for both water and sediment were present (*p* > 0.05). No significant differences were found when comparing the sediment time points (*p* > 0.05), while in the water samples, one significant difference was observed between treatment time points 1 and 7 (*p* ≤ 0.05).

### 3.3 β‐Diversity

**FIGURE 1:**
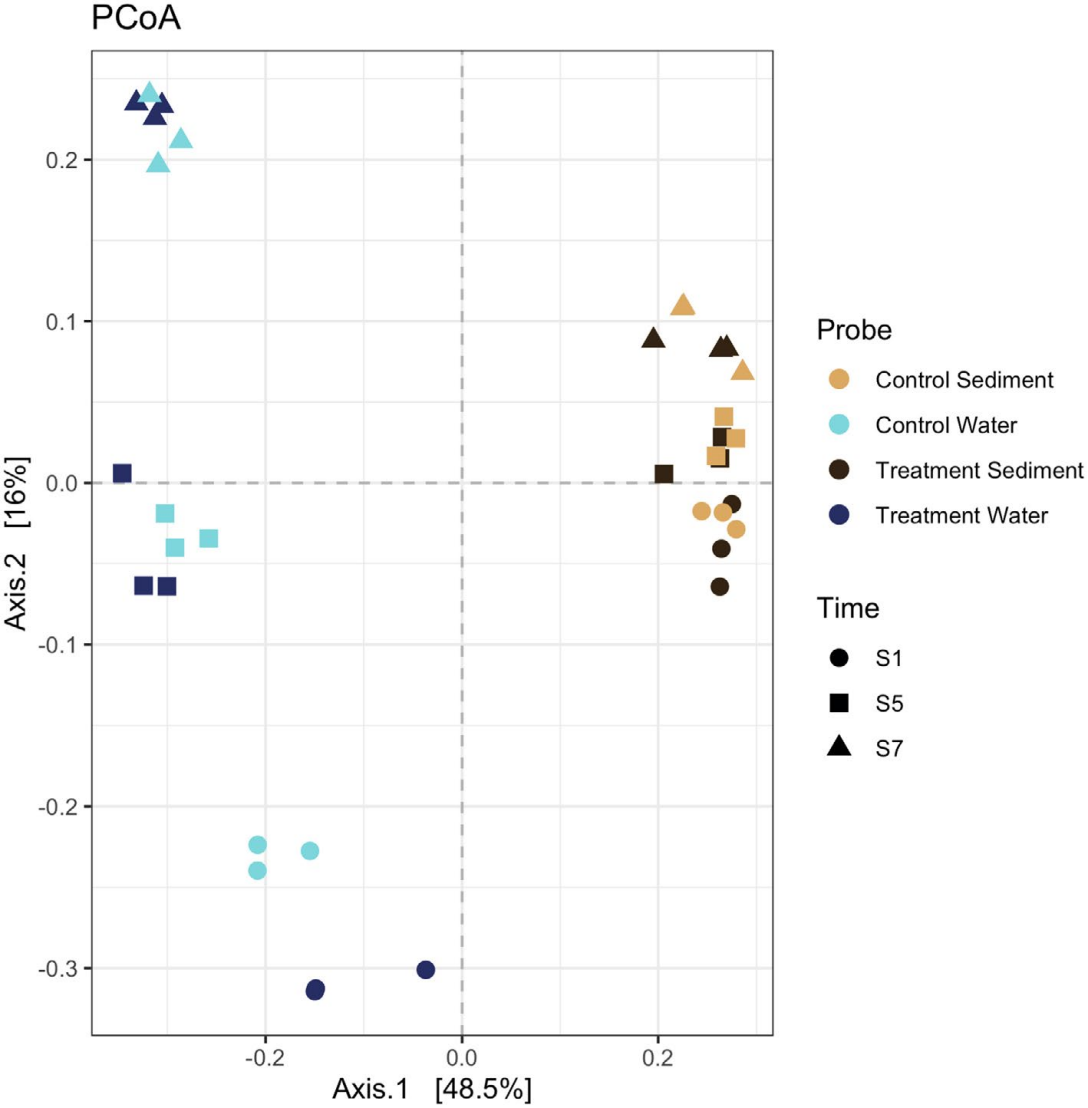
Principal Coordinates Analysis (PCoA). Samples are grouped as sediment control (light brown), water control (light blue), sediment treatment (dark brown), and water treatment (dark blue). Sampling times are indicated by different symbols: S1 = circle, S5 = square, and S7 = triangle.

Water hosts a distinctly different microbial community compared to sediment (Figure 1; *p* ≤ 0.05), a finding strongly supported by hierarchical clustering (Figure S5). In the PCoA, Axis 1 clearly separates samples by habitat, highlighting the strong compositional distinction between sediment and water. Axis 2, in turn, reflects temporal changes within habitats, indicating that time is a key factor influencing community structure. When comparing control and treatment samples within the water group, a distinct separation was observed at each sampling time point, showing clear differences between treatment and control (*p* ≤ 0.05). In contrast, this pattern was not evident in sediment samples, where no significant differences were found between treatment and control (*p* > 0.05). In totally 499 different OTUs were differentially abundant, when comparing treatment and controls overall (Table S1, *p* < 0.05). In detail 408 OTUs were differentially abundant when focusing on the water column (treatment vs. control) and 169 OTUs were different when focusing on sediment (treatment vs. control).

However, both habitats showed significant differences between sampling time points (*p* ≤ 0.05), with temporal variation emerging as a major driver of within‐habitat clustering. This effect was more pronounced in water samples than in sediment (Figure 1; see also Figure S5). These differences were generally more obvious in water samples, while less pronounced in sediment samples when examining taxonomic composition at the supergroup level (Figure S7).

### 3.4 OTU Overlap Between Habitats and Treatments

**FIGURE 2:**
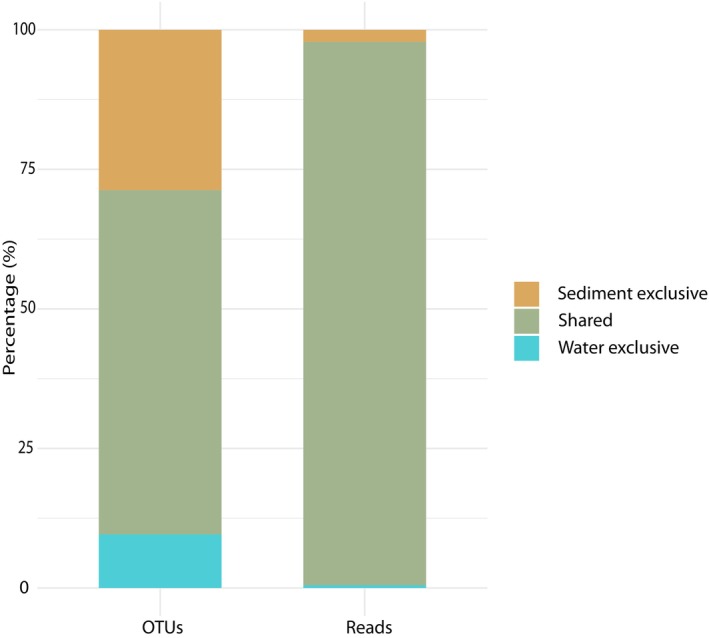
Distribution of OTUs (left) and reads (right), indicating whether they were found exclusively in one habitat or shared between habitats.

OTU analysis revealed distinct community compositions in sediment and water samples, comprising both habitat‐specific OTUs as well as a shared fraction present in both environments. Specifically, 1207 OTUs (28.96%) were unique to sediment, 400 OTUs (9.59%) were unique to water, and 2562 OTUs (61.45%) were shared between the two habitats (Figure 2). When read abundances were considered, however, this pattern shifted markedly: the vast majority of sequencing reads originated from shared OTUs (97.35%), indicating that although many OTUs were habitat‐specific, their relative abundances were rather low (Figure S6). OTUs exclusively found in sediment represented 2.16% of the total reads, whereas OTUs unique to water contributed 0.49%, further emphasizing the dominance of shared OTUs in terms of abundance.

When focusing on the OTU distribution between treatment and control we found that 101 OTUs (2.4%) were exclusively present in the controls, 3826 OTUs (91.8%) were found in the overlap of control and treatment, and 242 (5.8%) OTUs were exclusive for treated wastewater (Figure S9).

Even though the fraction of shared OTUs between water and sediment is high, most of these shared OTUs show clear preferences for either one of the habitat types. Most of the overlapping OTUs preferred the water habitat (53.25%). On the other hand, fewer shared OTUs showed preferences for sediment habitat (46.75%). When taking the read abundances of the OTUs into account, we found that in general, most shared OTUs had low or moderate read abundance and were found more prominently in both habitats. In addition, OTUs with higher read abundances showed stronger preferences for a specific habitat type and could be more clearly associated with either water or sediment.

### 3.5 Functional Composition

In total 14.4% of OTUs were assigned to parasites, 44.4% to consumers, 19.3% to mixotrophs, and 14.9% to phototrophic organisms (Table S2). When assigning trophic levels to the individual OTUs, 7.0% could not be assigned to a specific nutrition mode and were marked as “not identified.”

**FIGURE 3:**
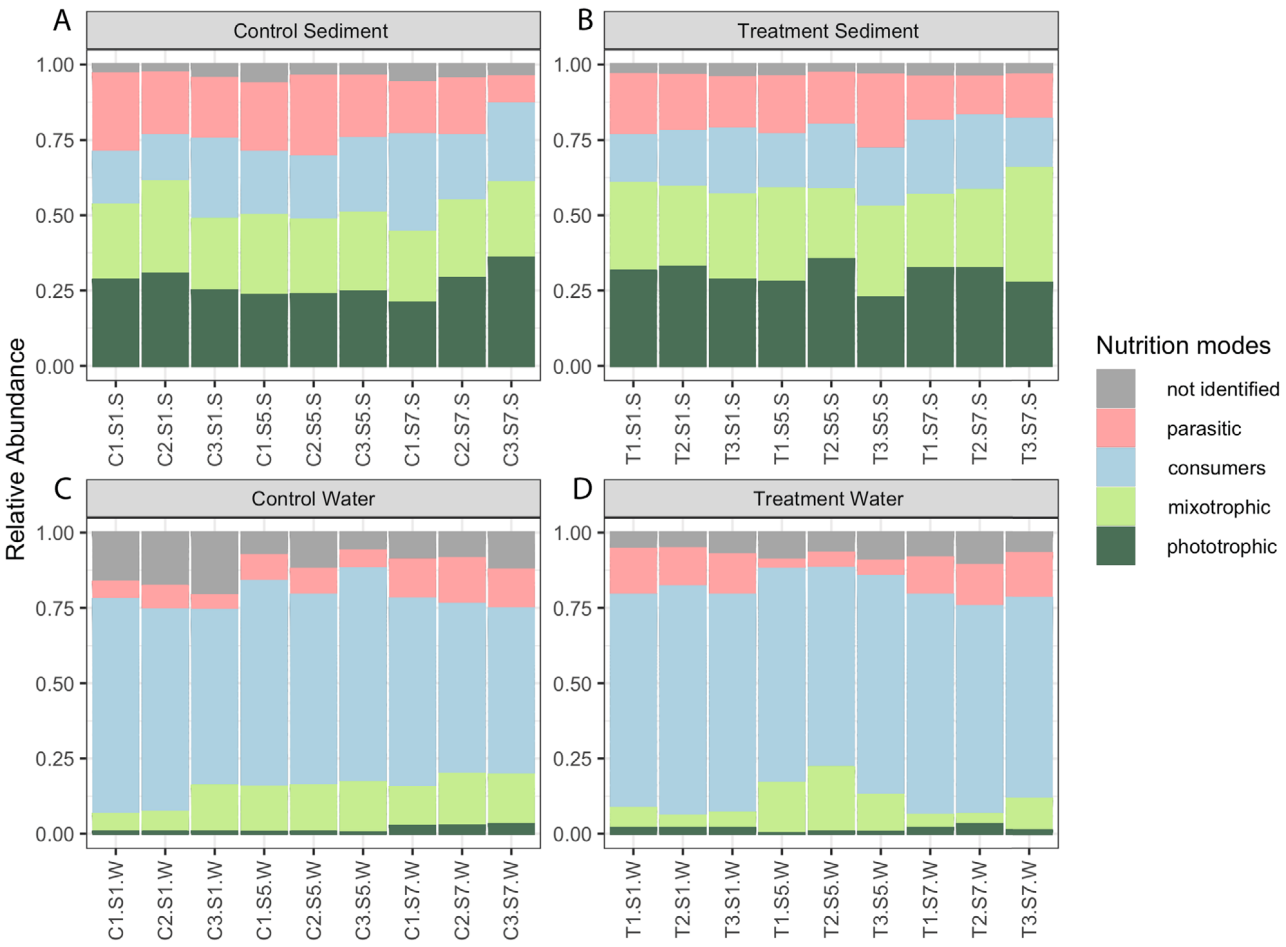
Stacked bar plots showing relative abundances of the nutrition modes in (A) the sediment control, (B) the water control, (C) the sediment treatment and (D) the water treatment over the whole sampling period. Nutrition modes were divided into parasites (red), consumers (blue), mixotrophs (purple) and phototrophs (green), as well as unidentified (gray).

In general, the percentage of unidentified organisms was higher in water than it was in sediment. Consumers were more abundant in water than in sediment making up 70.8% (±3.1%) in “water treatment,” 63.7% (±6.1%) in “water control,” 20% (±3.3%) in “sediment treatment” and 22.9% (±5.2%) in “sediment control.” Water included a lower number of phototrophic organisms (control = 1.3% (±1.1%), treatment = 1.4% (±0.9%)) while phototrophic organisms were more abundant in sediment, where they made up 30.1% (±3.8%) in treatment and 26.8% (±4.5%) in the control. Mixotrophic organisms made up a relatively small percentage in water (11.4%). In water treatment they made up 9.4% (±6.3%) and in water control 13.5% (±4.3%). However, they were the second most abundant in sediment with 27.1% in total. In sediment treatment mixotrophic organisms made up 28.6% (±4.4%) and in sediment control 25.7% (±2.1%). Parasites were more frequent in sediments (18.9%) than in water (9.9%), namely 17.7% (±3.5%) in sediment treatment, 20.3% (±5.2%) in sediment control, 10.6% (±4.8%) in water treatment and 9.2% (±3.7%) in water control (Figure 3).

### 3.6 Indicator OTUs

A total of 99 OTUs that were indicative for either one of the samples were identified (*p* < 0.05) (Table S3). In detail, 30 OTUs for “sediment treatment,” 64 OTUs for “water treatment,” nine OTUs for “sediment control” and seven OTUs for “water control.” In total, nine species were indicative for both “sediment treatment” and “water treatment.” No such overlap was identified for the control. Out of the 99 indicative taxa, 14 species could be taxonomically identified to the species level and cross‐referenced. All of these were indicator OTUs for “sediment treatment,” “water treatment” or both. In “sediment treatment” they were namely *Cercomonas braziliensis* (Cercozoa), *Euglypha acanthophora* (Cercozoa), *Euplotes elegans* (Ciliophora), *Heterobasidion parviporum* (Basidiomycota), *Nanofrustulum shiloi* (Bacillariophyceae), and *Paraphysomonas longispina* (Chrysophyceae). In “water treatment” the taxonomically identifiable indicator OTUs were *Apoikiospumella mondseeiensis* (Chrysophyceae), *Haematococcus pluvialis* (Chlorophyta), *Paraphysomonas vulgaris* (Chrysophyceae), *Rhogostoma minus* (Cercozoa), *Stygamoeba regulata* (Ancyromonadida), and *Uronemella filificum* (Ciliophora). We also found two OTUs that were indicative for both “sediment treatment” and “water treatment.” These were namely *Trichosporon cutaneum* (Basidiomycota) and *Paraphysomonas variosa* (Chrysophyceae).

### 3.7 Treated Wastewater Exclusive

**FIGURE 4:**
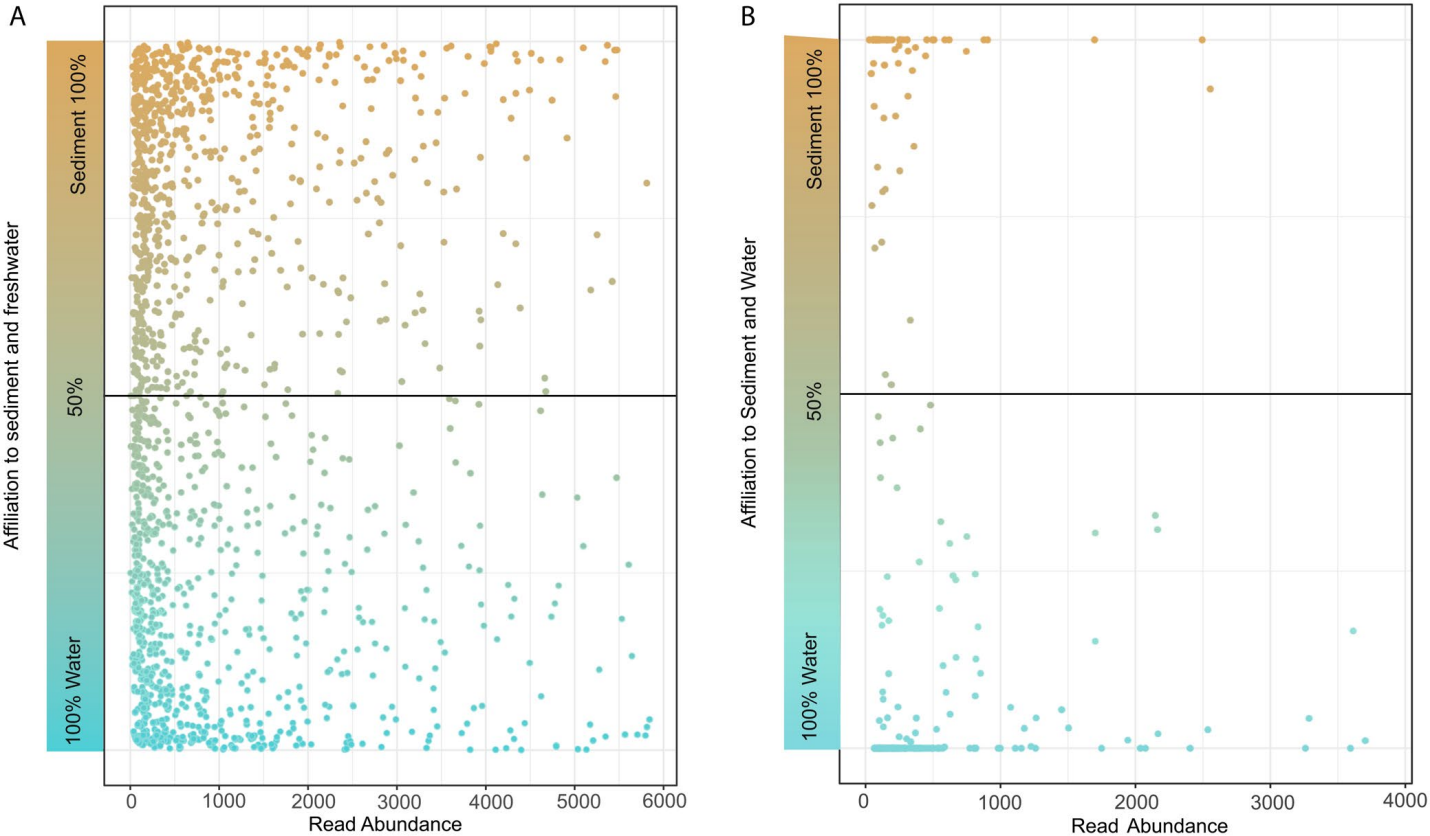
OTU abundance distribution patterns across water (blue) and sediment (brown) for shared OTUs. The *x*‐axis shows the number of reads, while the *y*‐axis indicates the affiliation to water or sediment. OTUs positioned along the 50/50 line indicate no habitat selectivity. Panel (A) shows the distribution of all shared OTUs with a cutoff at 80% of OTUs (for clarity, only the lower 80% in terms of abundance are shown; the full distribution is available in Figure S8). Panel (B) displays the distribution of 242 OTUs exclusively found in treated wastewater, with a cutoff at 90% of OTUs.

In total, 242 OTUs were exclusive for treated wastewater and were found exclusively in “water treatment” and “sediment treatment.” When looking at the general distribution of these OTUs, we found 41.7% in water, while 16.6% were found in sediment and 41.7% were shared (Figure S5). To analyze the distribution of shared OTUs between habitats, we assessed whether individual OTUs were more abundant in water or sediment (Figure 4B). In total, 91.5% of reads showed a preference for water, while 8.5% were more abundant in sediment. On the OTU level, 71.7% were associated with rather water and 28.3% with rather sediment. Most of the shared OTUs had relatively low read abundances (mean = 5440 ± 31,050).

### 3.8 Taxonomic Affiliation of OTUs Exclusive for Treated Wastewater

Analysis of the taxonomy of treatment‐exclusive OTUs (Table S4) showed that the majority of reads belonged to taxa affiliated with Gyrista (48.3%), a heterotrophic subgroup of Stramenopiles that includes Ochrophyta. Another major portion of the treatment‐exclusive taxa was assigned to Chrysophyceae (36.7%). Euglenozoa represented the third most abundant group (3.3%), while smaller proportions were attributed to Ciliophora (3.1%) and Ascomycota (1.9%). The high relative abundance of Chrysophyceae and Gyrista resulted primarily from a small number of OTUs with disproportionately high read counts. For Chrysophyceae, the three dominant OTUs accounted for 17.1%, 8.9%, and 5.6%. Similarly, the main OTUs assigned to Gyrista contributed 20.6%, 18.3%, and 3.5% of the treatment‐exclusive read abundances. However, these six OTUs could not be identified to a lower taxonomic level due to low bootstrap support.

## 4 Discussion

### 4.1 Significance

Freshwater ecosystems are under increasing pressure from human activities, particularly through the introduction of treated wastewater, which can profoundly alter microbial communities and ecosystem functions. Microeukaryotes, as key drivers of nutrient cycling and energy flow, serve as sensitive indicators of these changes. However, it remains unclear how microeukaryotic communities in different freshwater habitats—namely sediments and the water column—respond to such stressors.

In this study, we investigated how treated wastewater affects microeukaryotic diversity and community composition in both sediment and freshwater habitats using controlled mesocosm experiments over a 10‐day period. We specifically compared the responses of these two interconnected yet ecologically distinct habitats and examined their taxonomic overlap.

Our findings show that sediment microeukaryotic communities maintain higher diversity, while water column communities exhibit more pronounced compositional changes following the introduction of treated wastewater. These differences reflect the distinct community compositions and ecological characteristics of sediments and the water column. The introduction of treated wastewater primarily disrupts the water column, and the interaction between treated wastewater and sediments occurs mainly at the sediment surface, making stronger effects less likely. Due to their habitat characteristics and limited exposure to treated wastewater, sediments are less affected, and no firm conclusions about their intrinsic resilience can be drawn. However, they may potentially act as stabilizing components in impacted freshwater ecosystems.

### 4.2 Community Differentiation Between Sediment and Water Habitats

While the distinctness and overlap of microbial communities between terrestrial and aquatic systems has gained increasing attention (Sieber et al. 2020; Singer et al. 2021), sediments—despite their ecological importance—remain comparatively understudied. Our study now demonstrates that sediments harbor significantly higher diversity than freshwater habitats (Figures S3 and S4), mirroring patterns previously observed in marine systems (Schoenle et al. 2021; Wu et al. 2023) and extending these findings to freshwater environments. This higher diversity can be attributed to the fine particulate nature of sediments, which leads to a large surface‐to‐volume ratio (Parker et al. 2018) and their greater habitat heterogeneity providing numerous microhabitats that support diverse species assemblages. Furthermore, sediments experience less variable abiotic conditions compared to freshwater, which is subject to fluctuating light, turbidity, and flow regimes that can limit species coexistence and favor a few specialized taxa (Göransson et al. 2013; Myers et al. 2024; Nolte et al. 2010; Woodward et al. 2010).

Most variation in our data was explained by habitat type, highlighting the distinct nature of sediment and water column communities (Figure 1). Temporal variation also contributed significantly, with freshwater communities showing stronger compositional changes over time than sediments, a pattern reported previously in mesocosm systems (Graupner et al. 2017). In addition, we observed a general decline in richness and effective number of species over time (Figures S3 and S4), consistent with previous observations on phytoplankton communities (Di Cavalho et al. 2023). This suggests that the pattern is not merely a mesocosm artifact, but reflects natural dynamics where communities adapt over time.

Despite these differences, potential exchange of taxa between habitats is documented and driven by processes such as sinking biomass, resting stages, vertical mixing, dispersal, and particle resuspension (DesRosiers et al. 2022; Han et al. 2024). Our findings reveal a high degree of overlap in OTU composition (61.5%) between these habitats (Figure 2), which is expected given their close ecological connection (Shi et al. 2020; Wu et al. 2023). This certainly depends on multiple factors, among others the height of the water column and the flow velocity, as shallow, fast‐moving water inevitably supports the detachment of benthic, sediment‐bound taxa. Whereas, on the other hand, standing waters or slowly flowing waters tend to favor the sinking of organisms from the water column onto the sediment.

However, the existence of shared OTUs between freshwater and sediment confirms that these habitats are not isolated. The observed overlap indicates a potential source–sink relationship, in which sediment can serve as a reservoir for microeukaryotic taxa. While sediments are well recognized as seed banks for eggs, plant seedlings, and marine protists (Brock et al. 2003; Sanyal et al. 2022), our findings indicate that they may play a similar role in freshwater microbial communities. This is ecologically meaningful, as sediments may act as microbial seedbanks, supporting the recovery of freshwater communities after disturbances such as droughts or temporary pollution. Furthermore, this implies that freshwater may also serve as an important source of organisms following local extinction events in the sediment or during successional processes. Thus, the presence of common species may enhance overall ecosystem resilience, allowing both habitats to maintain functional stability, particularly after environmental stress. Whether the organisms are active or inactive (e.g., cysts) is of secondary importance; even if species sink onto the sediment and persist in a resting state, for example within a potentially protective biofilm, they retain the capacity to be transported back into the water column and thereby act as microbial seeds. Thus, even when detected as environmental DNA—encompassing both active and inactive organisms—this does not substantially affect the ecological interpretation of shared OTUs as indicators of connectivity and resilience between sediment and freshwater habitats. Rather, this represents a strength of environmental DNA approaches, as the same ecologically relevant patterns may not be detectable using RNA‐based methods.

It should be noted, however, that the mesocosms represent circulating systems. This may increase the likelihood that OTUs are transported, especially from the sediment into the water column. However, this contradicts our results, as more shared OTUs exhibit a preference for freshwater, suggesting either passive drift from the water column to the sediment or active growth in the water with resting stages in the sediment and that resuspension was low under our slow‐flow conditions. Examining relative abundances, shared OTUs that were present in roughly equal proportions between habitats were generally of low abundance, indicating that these represent true habitat generalists with no strong habitat preference, which are rare. In contrast, shared OTUs with higher relative abundances tended to be more habitat‐specific (Figure 4). This is in line with findings for soil and freshwater systems (Sieber et al. 2020).

### 4.3 Distinct Nutritional Strategies Shape Sediment and Freshwater Microeukaryotic Communities

While the feeding behavior of especially protists has been studied extensively (Ma et al. 2022; Sanders 2011), differences in their nutrition modes between sediment and freshwater habitats remain largely unexplored. Our results demonstrate clear distinctions in the dominant trophic strategies of protists inhabiting these two environments.

Surprisingly, phototrophic organisms such as Chlorophyceae and Bacillariophyceae were abundant in sediment samples but represented only a small fraction of freshwater communities. Although this might seem counterintuitive—since phototrophs are typically more prevalent in the planktonic fraction (Korneva 2012; Singer et al. 2021)—similar patterns have been reported previously (Jäger et al. 2017; Yang et al. 2021). It was shown that within environments characterized by shallow water columns and slow flow, such as our mesocosms, benthic algae dominate and phototrophs often settle in sediments where sufficient light penetration allows continued photosynthetic activity (Bharti et al. 2017; Roeselers et al. 2008; Sanli et al. 2015; Zhang et al. 2023).

Sediments also featured a wide array of heterotrophic protists, mainly Cercozoa and ciliates, consistent with earlier findings indicating these groups' preference for sediment habitats (Gücker and Fischer 2003; Manru et al. 1988). Furthermore, parasitic protists, predominantly Apicomplexa, were frequently detected in sediments, supporting previous observations that soils and sediments serve as reservoirs for parasitic taxa (Gad et al. 2024; Mahé et al. 2017).

In contrast, freshwater habitats were dominated by heterotrophs, notably fungi from Basidiomycota, Ascomycota, and Chytridiomycota, an unexpected finding given the typically higher phototrophic abundance in water columns. However, similar fungal prevalence has been reported by Wu et al. in freshwater and sediment samples (Wu et al. 2023). Moreover, other heterotrophic groups such as ciliates, choanoflagellates, and Euglenozoa were more abundant in freshwater, reflecting their adaptation to environments with high organic matter and bacterial productivity (Madoni 1994; Sultana et al. 2024; Weitere and Arndt 2002).

### 4.4 Response of Protist Communities to Treated Wastewater in Sediment and Freshwater Habitats

Treated wastewater (TWW) represents a widespread anthropogenic stressor impacting freshwater ecosystems (Guillet et al. 2019) by introducing a complex mixture of nutrients, contaminants, and microbes into recipient ecosystems, and yet its effects on the microbial community have not been studied in detail. Understanding how protist communities in both the water column and sediments respond to TWW is crucial for assessing ecosystem resilience and recovery potential. Our study reveals distinct responses in these habitats, highlighting the sediment's role as a microbial reservoir under stress.

In our experiment, the introduction of TWW had a pronounced impact on freshwater microeukaryotic communities, while sediment communities remained comparatively stable. The majority of OTUs (91.8%) were shared between treatment and control groups (Figure S6), indicating high baseline similarity. However, the TWW treatment resulted in a higher number of exclusive OTUs (242, 5.8%), suggesting that new taxa were introduced via the treated wastewater.

In line with our expectations, freshwater habitats showed a strong initial increase in species richness following TWW addition, reflecting an introduction of allochthonous taxa. This is consistent with earlier findings that highlight the introduction of novel taxa via treated effluents (Burdon et al. 2020; Stach et al. 2023). Interestingly, OTUs exclusively found in TWW‐treated mesocosms were predominantly associated with the freshwater habitat, reinforcing the idea that the water column is more vulnerable to stressor‐induced change (Nuy et al. 2018; Wu et al. 2023). In addition, previous studies have also reported that microbial communities within the water column progressively diverge under sustained anthropogenic stressor exposure (Boden et al. 2023; Graupner et al. 2017). In contrast, sediment richness remained largely unaffected, highlighting its greater buffering capacity and physical separation from direct treated wastewater exposure (Boeraș et al. 2024; Guo et al. 2022). On the one hand, this is logical, as primarily water was introduced into another water body. At the same time, however, we have shown that both habitats can function as mutual seed banks; therefore, it is only logical that organisms are also introduced from the water column into the sediment. Furthermore, benthic organisms are present in wastewater treatment plants, and although the particulate fraction is removed as efficiently as possible by sedimentation, some benthic organisms are inevitably discharged into receiving waters due to resuspension.

Whether these allochthonous taxa can also be considered invasive is difficult to determine and to generalize, as the differences they introduce decrease over time, and previous studies have shown that such introduced species tend to decline after initial entry (Stach et al. 2023; Sieber et al. 2023). Nonetheless, it is evident that these taxa can temporarily disrupt the ecosystem by altering community composition and, consequently, community interactions. Even if they do not establish permanently, the repeated pulses of treated wastewater ensure that these taxa are continually introduced, thereby affecting ecological dynamics on a recurring basis. This highlights the importance of considering both transient and persistent effects when evaluating the ecological impact of treated wastewater on freshwater communities, as it is likely that at least some allochthonous taxa may be pathogenic or invasive.

Although nutrient concentrations in TWW are regulated, inputs often exceed background levels in receiving waters and can induce community shifts, particularly among microeukaryotes (Barbosa et al. 2022; Liu et al. 2022). Sediments, however, may experience fewer changes due to naturally higher nutrient loads and slower diffusion dynamics. Therefore, sediments may function more as filters or sinks, where newly introduced species fail to establish due to environmental constraints (Guo et al. 2022).

As the experiment progressed, freshwater communities in treatment and control mesocosms began to converge, suggesting a potential recovery of the microbial community. In contrast, no similar compositional changes were observed in the sediment communities, highlighting again their resilience and temporal stability in response to TWW (Ruprecht et al. 2020). Nevertheless, given the limited duration of 10 days, possible long‐term impacts beyond this timeframe cannot be assessed and require future study.

Our findings clearly demonstrate that habitat type was the strongest explanatory factor in community composition, with freshwater communities more strongly affected by both TWW and temporal dynamics. This supports the view that sediment communities are more resistant to short‐term perturbations and may act as microbial reservoirs buffering environmental stress. This increased resilience may be attributed to their biofilm‐based organization, which offers structural protection and functional redundancy, thus enabling greater temporal stability in response to treated wastewater input (Guo et al. 2022; Roeselers et al. 2008).

Together, our findings highlight the differential susceptibility of freshwater and sediment microeukaryotic communities to treated wastewater. While freshwater assemblages are sensitive and responsive, sediment communities appear comparatively robust—likely due to physical separation, biofilm formation, and higher habitat complexity. These insights are particularly relevant for ecosystem monitoring and wastewater management, emphasizing the need to focus on water column communities as sensitive indicators of anthropogenic disturbance, while acknowledging the stabilizing role of sediments in preserving microbial diversity.

### 4.5 Indicator Taxa Reveal Treated Wastewater Exposure in Aquatic Systems

Beyond these community‐level patterns, the identification of specific taxa indicative of treated wastewater input provides an additional tool for environmental monitoring and assessment.

From a monitoring perspective, the habitat‐specific occurrence of these indicator taxa suggests that they could be used to detect the presence and spatial extent of treated wastewater influence in freshwater systems. Changes in the presence or abundance of these taxa could therefore be used to track temporal changes in wastewater influence, for example before and after the implementation of wastewater treatment or management measures. In this context, indicator taxa may support the indirect assessment of treatment performance or management effectiveness, rather than providing a direct measure of treatment efficiency. Ciliates are well known to include suitable indicator species for wastewater and have been used to assess various stages of wastewater treatment (Foissner 2016). Our results suggest, however, that a broader range of taxa should be considered when assessing the impact of treated wastewater in freshwater environments. Although species‐level taxonomic resolution was not always possible for all OTUs, a total of 14 indicative taxa could be reliably identified, of which only 5 were previously associated with wastewater.

*Trichosporon cutaneum*, *Euplotes elegans*, and *Paraphysomonas variosa* were consistently associated with treated wastewater in both water and sediment samples. While the first two species were already known to be linked to wastewater environments (Dragičević et al. 2010; Schwarz et al. 2007; Tomaru 2002; Xiong et al. 2015), *P*. *variosa* had not previously been described in this context.

Several taxa were exclusively indicative of treated wastewater in the water column, including *Rhogostoma minus*, *Haematococcus pluvialis* , *Paraphysomonas vulgaris*, *Apoikiospumella mondseeiensis*, *Stygamoeba regulata*, and *Uronemella filificum*. Of these, only *R*. *minus* and *H*. *pluvialis* have previously been associated with wastewater exposure (Aydin et al. 2022; Kang et al. 2006; Pohl et al. 2021).

In contrast, *Nanofrustulum shiloi*, *Paraphysomonas longispina*, *Heterobasidion parviporum*, *Cercomonas braziliensis*, and *Euglypha acanthophora* were found exclusively in sediments exposed to treated wastewater, with only *E*. *acanthophora* previously reported from such environments (Chouari et al. 2017; Jaromin‐Gleń et al. 2013).

Together, these findings highlight the potential of previously overlooked microeukaryotic taxa as sensitive and habitat‐specific indicators of treated wastewater, offering new tools for environmental monitoring and early detection of anthropogenic disturbance in freshwater ecosystems. Furthermore, it shows that despite the known and commonly used bioindicators, many—in our case more than two‐thirds—remain undescribed for specific habitats. Interestingly, these are not limited to classical indicator groups such as heterotrophic organisms (e.g., ciliates or amoebae), but also include organisms from other major taxonomic groups (e.g., Bacillariophyceae and Chlorophyceae). Accordingly, the overall potential of bioindicators is far from being fully realized. While the origin of many detected OTUs is likely linked to treated wastewater, this cannot be conclusively determined based on the current dataset. To generalize these findings and to expand the repertoire of indicative taxa, further investigations including diverse wastewater sources is needed. In particular, distinguishing between municipal and industrial effluents would be valuable, as the present study focused on municipal wastewater.

## 5 Conclusion

Our results demonstrate that, although freshwater and sediment are both aquatic habitats with overlapping microeukaryotic species pools, their communities differ substantially—not only in composition but also in their response to treated wastewater input. While the water column community exhibits a pronounced but temporary shift in composition—including the emergence of several previously unreported indicator species—the sediment community remains largely stable throughout the experiment. This suggests that structural and functional characteristics such as biofilm formation, spatial insulation, higher diversity, and habitat complexity may confer a buffering capacity to benthic communities. These findings underline the importance of including both habitat types in environmental assessments to avoid underestimating the ecological effects of treated wastewater. Moreover, the identification of 14 wastewater‐associated indicator taxa, including nine not previously linked to such environments, highlights the potential of microeukaryotic communities as sensitive bioindicators. However, given the limited temporal scale of this study, further research is needed to evaluate long‐term impacts and to assess how recurring or chronic exposure might alter microbial community dynamics and ecosystem functioning.

## Supporting information


**Figure S1:** Schematic setup of the AquaFlow mesocosms. A shows one of the mesocosms in detail. Within this picture A is the first water tank (270 L), B and C are the second and third water tank (40 L each). D and E are sediment channels (both 10 cm wide, 4 m and 2 m long). F is the cooling element. B shows the setup of all six mesocosm systems at their location in the greenhouses of the University of Duisburg‐Essen.

**Figure S2:** Rarefaction curves for (A) water and (B) sediment samples showing the abundance of reads on the *x*‐axis and the OTU number on the *y*‐axis.

**Figure S3:** Boxplot of α‐diversity showing OTU richness across different groups and time points. “Water Control” and “Sediment Control” represent all water and sediment samples, respectively, under control conditions, while “Water Treatment” and “Sediment Treatment” include all sampling time points under treatment conditions. The numbers (1, 5, 7) represent the sampling times (1 h, 4 days, 10 days).

**Figure S4:** Showing effective number of species across habitats and sampling points.

**Figure S5:** Hierarchical clustering of samples by Bray–Curtis Distance based on ward.d2, showing sediment control (light brown), sediment treatment (dark brown), water control (light blue) and water treatment (dark blue).

**Figure S6:** showing the distribution of OTUs and read abundances, between all control samples (including water and sediment) and all treatment samples (including water and sediment).

**Figure S7:** Stacked bar plots showing the distribution of relative abundances of taxonomic groups in (A) the sediment control (B) the water control (C) the treated sediment and (D) the treated water samples over the whole sampling period.

**Figure S8:** OTU abundance distribution patterns across water (blue) and sediment (brown) for shared OTUs. The *x*‐axis shows the number of reads, while the *y*‐axis indicates the affiliation to water or sediment. OTUs positioned along the 50/50 line indicate no habitat.

**Figure S9:** Venn‐Diagram showing the overlap between the water and sediment for the 242 OTUs which were exclusive for treated wastewater.

**Table S1:** Differentially abundant OTUs and their corresponding log_2_ fold changes. Only OTUs that were significantly differentially abundant (adjusted *p* < 0.05) and exhibited a log_2_ fold change greater than 2 were included. Values in the taxonomic path indicate *mothur* bootstrap confidence scores.

**Table S2:** Affiliation of individual groups and organisms to nutrition modes.

**Table S3:** Indicative OTUs for sediment treatment (S/T), sediment control (S/C), water treatment (W/T) and water control (W/C). The numbers after each taxonomic assignment represent bootstrap values.

**Table S4:** Showing the organismic groups that were found exclusively in treated wastewater and their relative proportion.

## Data Availability

The data that support the findings of this study are available in NCBI SRA at https://www.ncbi.nlm.nih.gov/sra, reference number PRJNA1019091. These data were derived from the following resources available in the public domain: https://www.ncbi.nlm.nih.gov/sra, https://www.ncbi.nlm.nih.gov/sra/?term=PRJNA1019091.
