# Supplementary material for: Freshwater and Sediment Host Distinct Yet Overlapping Microeukaryotic Communities, With Sediment Communities Less Impacted by Treated Wastewater
Source: J Eukaryot Microbiol. 2026 Feb 24;73(2):e70070. doi: 10.1111/jeu.70070 (PMC12932744; doi:10.1111/jeu.70070)
Supplement: Supplementary file 1 — Figure S1: Schematic setup of the AquaFlow mesocosms. A shows one of the mesocosms in detail. Within this picture A is the first water tank (270 L), B and C are the second and third water tank (40 L each). D and E are sediment channels (both 10 cm wide, 4 m and 2 m long). F is the cooling element. B shows the setup of all six mesocosm systems at their location in the greenhouses of the University of Duisburg‐Essen. [file JEU-73-e70070-s002.pdf]

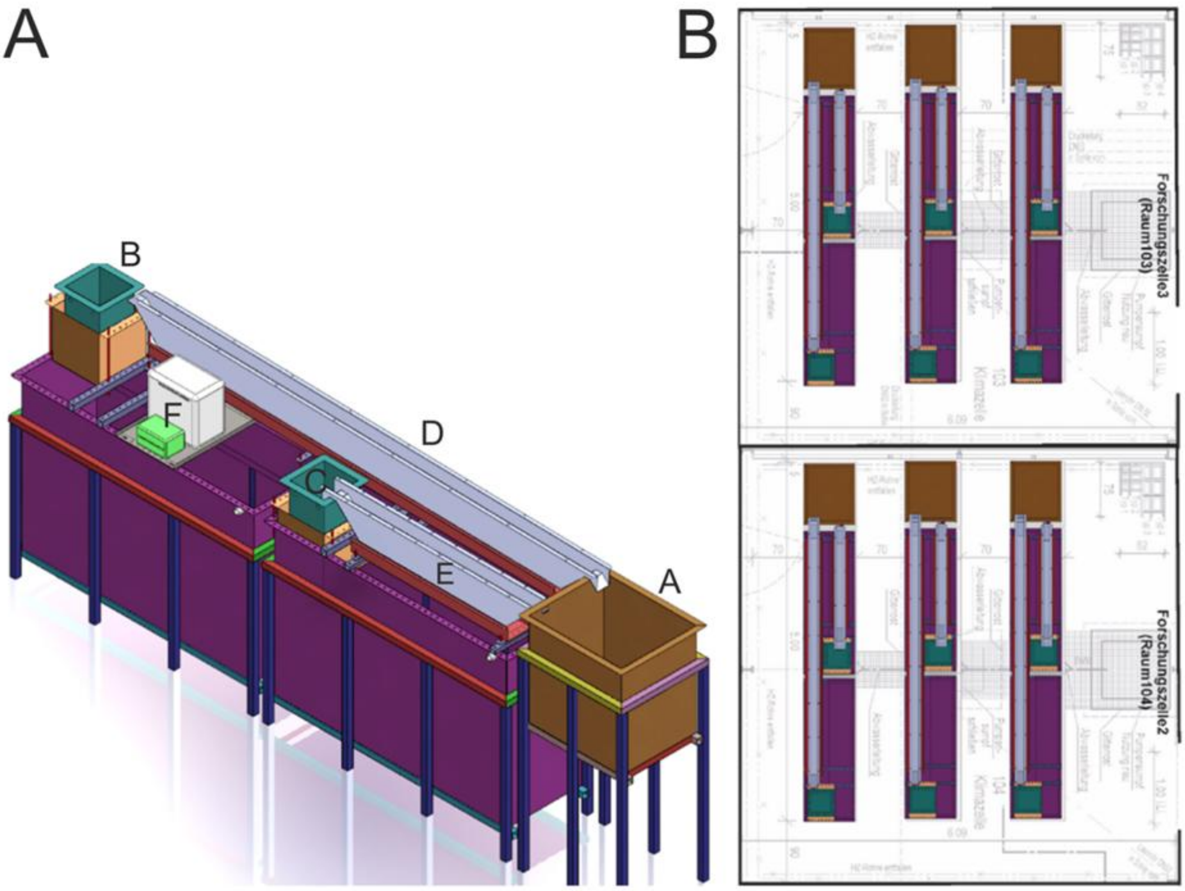
A
B
B
C
D
E
F
A
Forschungszelle3 (Raum103)
Forschungszelle2 (Raum104)
