# Supplementary figures and images for: Freshwater and Sediment Host Distinct Yet Overlapping Microeukaryotic Communities, With Sediment Communities Less Impacted by Treated Wastewater
Source: J Eukaryot Microbiol. 2026 Feb 24;73(2):e70070. doi: 10.1111/jeu.70070 (PMC12932744; doi:10.1111/jeu.70070)

Sediment

Water

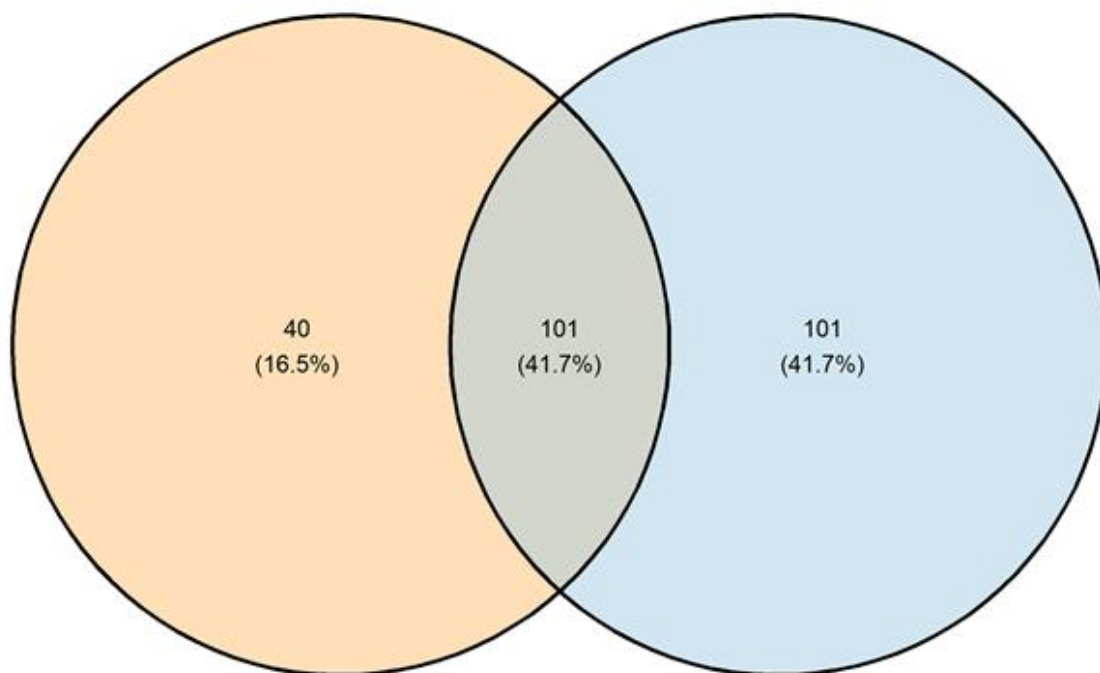

Supplement: Supplementary file 9 — Figure S9: Venn‐Diagram showing the overlap between the water and sediment for the 242 OTUs which were exclusive for treated wastewater. [file JEU-73-e70070-s003.pdf]
