# Supplementary material for: Freshwater and Sediment Host Distinct Yet Overlapping Microeukaryotic Communities, With Sediment Communities Less Impacted by Treated Wastewater
Source: J Eukaryot Microbiol. 2026 Feb 24;73(2):e70070. doi: 10.1111/jeu.70070 (PMC12932744; doi:10.1111/jeu.70070)
Supplement: Supplementary file 11 — Table S2: Affiliation of individual groups and organisms to nutrition modes. [file JEU-73-e70070-s008.pdf]

Organism affiliation	Nutrition mode
Acrispumella	consumers
Actinocephalidae	parasitic
Amoebozoa	consumers
Amphidiniopsidaceae	consumers
Ancoracysta	consumers
Ancyromonadida_Group-1_X	consumers
Anisonemidae	consumers
Apicomplexa	parasitic
Apoikiales	consumers
Bacillariophyceae	phototrophic
Bigyra	consumers
Bolidophyceae	phototrophic
Breviatea	consumers
Centroplasthelida	consumers
Cercozoa	parasitic
Chlorophyceae	mixotrophic
Chlorophyta	phototrophic
Chlorophyta_X	phototrophic
Choanoflagellata	consumers
Chromulina	mixotrophic
Chrysoamoebidaceae	mixotrophic
Chrysocapsaceae	mixotrophic
Chrysonephele	mixotrophic

Chrysosaccales	mixotrophic
Chytridiomycota	parasitic
Ciliophora	consumers
Coccidiniales	parasitic
Collodictyonidae	consumers
Colpodellida	consumers
Cornospumella	consumers
Cryptodifflugia	consumers
Cryptophyta	phototrophic
Cyclonexis	mixotrophic
Dinobryon	mixotrophic
Dinophysiales	mixotrophic
Diplonemidae	consumers
Epipyxis	mixotrophic
Euglenales	consumers
Euglenoidea	consumers
Euglenophyceae	phototrophic
Eustigmatophyceae	phototrophic
Flamella_pleistocenica;	consumers
Fungi	consumers
Gonyaulacales	mixotrophic
Gymnodiniales	mixotrophic
Gymnoxanthella	parasitic
Haplozoonales	parasitic

Haptophyta	phototrophic
Hartmannella	consumers
Heterolobosea	consumers
Hibberdiales	mixotrophic
Hydrurales	mixotrophic
Ichthyosporea	parasitic
Jakobida	consumers
Kathablepharidaceae	consumers
Kinetoplastea	consumers
Lentomonas	mixotrophic
Litostomatea	mixotrophic
Lophodiniales	mixotrophic
Mamiellophyceae	mixotrophic
Mastigamoeba	consumers
Mayorella	consumers
Metamonada	parasitic
Ochromonas	mixotrophic
Oikomonadaceae	parasitic
Oodiniaceae	parasitic
Oomycota	parasitic
Opalinata	parasitic
Parabodonida	parasitic
Paradermamoeba	consumers

Paramoeba	consumers
Paraphysomonas	consumers
Paravannella	consumers
Pedospumella	consumers
Peranemidae	consumers
Peridiniales	mixotrophic
Petalomonadida	consumers
Pfiesteriaceae	consumers
Planomonadidae_Group-2_X_sp.;	consumers
Poterioochromonas	mixotrophic
Poteriospumella	consumers
Prorocentrales	mixotrophic
Prymnesiophyceae	mixotrophic
Pseudoparamoeba	consumers
Pyramimonadophyceae	mixotrophic
Raphidophyceae	phototrophic
Reclinomonas_americana	mixotrophic
Rhizaria	consumers
Rigifilida	consumers
Rotosphaerida	consumers
Saccamoeba	consumers

Schizopyrenida	parasitic
Segregatospumellales	consumers
Spirotrichea	mixotrophic
Stramenopiles	consumers
Stygamoeba_regulata	consumers
Suessiaceae	phototrophic
Spumella	consumers
Symbiodiniaceae	parasitic
Syndiniales	parasitic
Synuraceae	mixotrophic
Synurales	phototrophic
Telonemia	consumers
Thoracosphaerales	parasitic
Trebouxiophyceae	mixotrophic
Trypanosomatida	parasitic
Uroglena	mixotrophic
Uroglenopsis	mixotrophic
Urostipulosphaera	mixotrophic
Vexillifera	consumers
