# Supplementary material for: Freshwater and Sediment Host Distinct Yet Overlapping Microeukaryotic Communities, With Sediment Communities Less Impacted by Treated Wastewater
Source: J Eukaryot Microbiol. 2026 Feb 24;73(2):e70070. doi: 10.1111/jeu.70070 (PMC12932744; doi:10.1111/jeu.70070)
Supplement: Supplementary file 12 — Table S3: Indicative OTUs for sediment treatment (S/T), sediment control (S/C), water treatment (W/T) and water control (W/C). The numbers after each taxonomic assignment represent bootstrap values. [file JEU-73-e70070-s001.pdf]

OTU-ID	Taxonomy	S/T p<0.05	S/C p<0.05	W/T p<0.05	W/C p<0.05
N259197_290853	Eukaryota(100);TSAR(100);Stramenopiles(100);Gyrista(100);Coscinodiscophyceae(44);Aulacoseirales(44);Aulacoseiraceae(44);Aulacoseira(44);Aulacoseira_baicalensis(18);	X		X	
N366615_63383	Eukaryota(100);Obazoa(100);Opisthokonta(100);Fungi(97);Ascomycota(96);Pezizomycotina(82);Pezizomycetes(52);Pyronema(12);Pyronema_omphalodes(12);	X		X	
N103686_5547	Eukaryota(100);Obazoa(89);Opisthokonta(89);Fungi(71);Opisthosporidia(30);Microsporida(30);Microsporida_X(30);Paramicrosporidium(30);Paramicrosporidium_saccamoebae(30);	X			
N293811_204	Eukaryota(100);Obazoa(100);Opisthokonta(100);Ichthyosporea(88);Dermocystida(88);Rhynosporidae(88);Rhynosporidae_X(88);Amphibiocystidium(45);Amphibiocystidium_sp.(22);	X		X	
N101273_67574	Eukaryota(100);TSAR(100);Stramenopiles(100);Gyrista(100);Chrysophyceae(74);Ochromonadales(61);Ochromonadales_clade-VI(35);Ochromonadales_clade-VI_X(35);Ochromonadales_clade-VI_X_sp.(35);	X		X	
N211848_21191	Eukaryota(100);Obazoa(100);Opisthokonta(100);Fungi(100);Basidiomycota(99);Agaricomycotina(98);Tremellomycetes(98);Trichosporon(98);Trichosporon_cutaneum(71);	X		X	
N94292_2127	Eukaryota(100);TSAR(100);Stramenopiles(99);Gyrista(99);Coscinodiscophyceae(57);Aulacoseirales(54);Aulacoseiraceae(54);Aulacoseira(54);Aulacoseira_nyassensis(45);	X			
N285906_2069	Eukaryota(100);TSAR(100);Stramenopiles(100);Gyrista(100);Bacillariophyceae(100);Thalassiophysales(21);Catenulaceae(21);Amphora(21);Amphora_coffeaeformis(21);	X		X	
N327073_1334	Eukaryota(100);TSAR(92);Rhizaria(92);Cercozoa(92);FilosaSarcomonadea(87);Cercomonadida(87);Cercomonadidae(87);Cercomonas(82);Cercomonas_braziliensis(62);	X			
N303332_912	Eukaryota(100);Obazoa(100);Opisthokonta(100);Ichthyosporea(87);Dermocystida(87);Rhynosporidae(87);Rhynosporidae_X(87);Amphibiocystidium(49);Amphibiocystidium_sp.(25);	X			
N117354_112602	Eukaryota(100);TSAR(100);Alveolata(100);Ciliophora(100);Spirotrichea(100);Euplotia(100);Euplotidae(100);Euplotes(100);Euplotes_elegans(100);	X			
N64739_4845	Eukaryota(100);Obazoa(100);Opisthokonta(100);Fungi(100);Ascomycota(99);Saccharomycotina(98);Saccharomycetales(96);Galactomyces(59);Galactomyces_geotrichum(48);	X			
N372694_497	Eukaryota(100);Obazoa(100);Opisthokonta(100);Fungi(100);Basidiomycota(100);Agaricomycotina(100);Agaricomycetes(100);Heterobasidion(100);Heterobasidion_parviporum(100);	X			
N292921_13837	Eukaryota(100);TSAR(99);Rhizaria(99);Cercozoa(99);Filosa-Imbricatea(99);Euglyphida(99);Euglyphidae(99);Euglypha(99);Euglypha_acanthophora(98);	X			
N175835_1015	Eukaryota(99);Obazoa(89);Opisthokonta(89);Fungi(75);Opisthosporidia(43);Microsporida(43);Microsporida_X(43);Paramicrosporidium(43);Paramicrosporidium_saccamoebae(43);	X		X	
N60915_467	Eukaryota(98);Amoebozoa(76);Tubulinea(72);Tubulinea_X(72);Elardia(72);Euamoebida(72);Vermamoebidae(28);Hartmannella(28);Hartmannella_cantabrigiensis(28);	X			
N66251_417	Eukaryota(100);TSAR(94);Alveolata(94);Ciliophora(93);Prostomatea_1(25);Prostomatea_1_X(25);Colepidae(25);Apocoleps(11);Apocoleps_orientalis(6);	X			
N15907_386	Eukaryota(100);TSAR(67);Rhizaria(66);Cercozoa(66);Filosa-Imbricatea(39);Thaumatomonadida(15);Esquamulidae(15);Esquamula(15);Esquamula_lacrimiformis(15);	X			
N239840_2109	Eukaryota(100);Obazoa(95);Opisthokonta(95);Fungi(83);Opisthosporidia(43);Microsporida(43);Microsporida_X(43);Paramicrosporidium(43);Paramicrosporidium_saccamoebae(43);	X		X	
N151658_443	Eukaryota(100);TSAR(93);Stramenopiles(93);Gyrista(93);Coscinodiscophyceae(32);Aulacoseirales(30);Aulacoseiraceae(30);Aulacoseira(30);Aulacoseira_baicalensis(12);	X			
N294745_339139	Eukaryota(100);TSAR(93);Stramenopiles(91);Gyrista(89);Phaeothamniophyceae(14);Phaeothamniales(14);Phaeothamniales_X(14);Phaeoschizochlamys(14);Phaeoschizochlamys_santosii(14);	X			
N259983_14029	Eukaryota(100);TSAR(100);Stramenopiles(100);Gyrista(100);Chrysophyceae(98);Paraphysomonadales(88);Paraphysomonadaceae(88);Paraphysomonas(88);Paraphysomonas_variosa(72);	X		X	
N189888_9709	Eukaryota(100);TSAR(100);Stramenopiles(100);Gyrista(100);Chrysophyceae(97);Paraphysomonadales(94);Paraphysomonadaceae(94);Paraphysomonas(94);Paraphysomonas_longispina(62);	X			
N59059_1545	Eukaryota(100);Obazoa(96);Opisthokonta(96);Fungi(76);Opisthosporidia(25);Microsporida(25);Microsporida_X(25);Paramicrosporidium(25);Paramicrosporidium_saccamoebae(25);	X			
N381048_859	Eukaryota(100);TSAR(100);Stramenopiles(100);Gyrista(100);Bacillariophyceae(93);Naviculales(91);Sellaphoraceae(88);Sellaphora(88);Sellaphora_laevissima(49);	X			
N423019_679	Eukaryota(100);TSAR(97);Stramenopiles(97);Gyrista(97);Coscinodiscophyceae(30);Aulacoseirales(30);Aulacoseiraceae(30);Aulacoseira(30);Aulacoseira_baicalensis(12);	X			
N284117_650	Eukaryota(99);TSAR(91);Stramenopiles(91);Gyrista(89);Bacillariophyceae(65);Naviculales(37);Berkeleyaceae(19);Climaconeis(19);Climaconeis_scalaris(19);	X			
N15907_386	Eukaryota(100);TSAR(67);Rhizaria(66);Cercozoa(66);Filosa-Imbricatea(39);Thaumatomonadida(15);Esquamulidae(15);Esquamula(15);Esquamula_lacrimiformis(15);	X			

N222044_468	Eukaryota(100);TSAR(99);Stramenopiles(99);Gyrista(99);Bacillariophyceae(98);Fragilariales(86);Staurosiraceae(84);Nanofrustulum(73);Nanofrustulum_shiloi(73);	X			
N74179_242	Eukaryota(100);TSAR(99);Stramenopiles(99);Gyrista(99);Eustigmatophyceae(56);Eustigmatophyceae_X(56);Eustigmatophyceae_XX(56);Pseudocharaciopsis(31);Pseudocharaciopsis_ovalis(31);	X			
N16919_148	Eukaryota(100);Amoebozoa(95);Tubulinea(94);Tubulinea_X(94);Elardia(94);Euamoebida(94);Hartmannellidae(80);Copromyxa(74);Copromyxa_microcystidis(65);		X		
N347124_1730	Eukaryota(100);TSAR(100);Stramenopiles(100);Gyrista(100);Xanthophyceae(100);Xanthophyceae_X(100);Xanthophyceae_XX(100);Vaucheria(100);Vaucheria_bursata(100);		X		
N171296_311	Eukaryota(100);Obazoa(100);Opisthokonta(100);Ichthyosporea(80);Dermocystida(80);Rhynosporidae(80);Rhynosporidae_X(80);Dermocystidium(71);Dermocystidium_sp.(71);		X		
N192182_297	Eukaryota(100);TSAR(75);Rhizaria(74);Cercozoa(74);Filosa-Imbricatea(74);Euglyphida(74);Euglyphidae(69);Euglypha(31);Euglypha_filifera(27);		X		
N337575_287	Eukaryota(99);Amoebozoa(87);Discosea(84);Discosea_X(84);Centramoebia(84);Himatismenida(84);Cochliopodiidae(84);Cochliopodium(84);Cochliopodium_kieliense(38);		X		
N311502_244	Eukaryota(100);TSAR(96);Rhizaria(90);Cercozoa(90);Endomyxa(90);Vampyrellida(90);Leptophryidae(90);Vernalophrys(52);Vernalophrys_algivore(52);		X		
N216190_161	Eukaryota(98);TSAR(67);Rhizaria(60);Cercozoa(60);Endomyxa(60);Vampyrellida(60);Sey055-lineage(37);Sey055-lineage_X(37);Sey055-lineage_X_sp.(37);		X		
N237703_358	Eukaryota(100);Archaeplastida(100);Chlorophyta(100);Chlorophyta_X(100);Ulvophyceae(100);Ulvales-relatives(100);Ulvales-relatives_X(100);Desmochloris(92);Desmochloris_halophila(56);		X		
N422854_102	Eukaryota(100);Obazoa(99);Opisthokonta(99);Fungi(97);Chytridiomycota(52);Rhizophydiales(47);Rhizophydiales_X(27);Rhizophydiales_XX(27);Rhizophydiales_XX_sp.(27);		X		
N125294_810	Eukaryota(100);Obazoa(91);Opisthokonta(91);Fungi(72);Basidiomycota(34);Pucciniomycotina(25);Cystobasidiomycetes(16);Sporobolomyces(15);Sporobolomyces_ruber(12);				X
N87944_8069	Eukaryota(100);Obazoa(96);Opisthokonta(96);Fungi(65);Aphelidiomycota(10);Aphelidiales(10);Aphelidiaceae(10);Amoeboaphelidium(10);Amoeboaphelidium_protococcarum(10);				X
N15032_115	Eukaryota(99);TSAR(81);Stramenopiles(52);Bigyra(25);Opalozoa(25);Opalinata(25);Nucleohelea(25);Actinosphaerium(25);Actinosphaerium_eichhornii(25);				X
N142855_2338	Eukaryota(100);Obazoa(100);Opisthokonta(100);Fungi(90);Ascomycota(72);Saccharomycotina(60);Saccharomycetales(52);Candida(46);Candida_galacta(24);				X
N409906_377	Eukaryota(100);Excavata(78);Discoba(75);Euglenozoa(73);Kinetoplastea(45);Prokinetoplastida(43);Ichthyobodonidae(43);Ichthyobodo(34);Ichthyobodo_hippoglossi(4);				X
N77143_389	Eukaryota(92);TSAR(69);Stramenopiles(45);Bigyra(32);Opalozoa(32);Opalinata(32);Nucleohelea(19);Actinosphaerium(19);Actinosphaerium_eichhornii(19);				X
N277607_159	Eukaryota(97);Obazoa(89);Opisthokonta(89);Fungi(68);Chytridiomycota(19);Rhizophydiales(11);Gorgonomycetaceae(10);Gorgonomyces(10);Gorgonomyces_haynaldii(10);				X
N17630_17422	Eukaryota(96);TSAR(82);Alveolata(79);Ciliophora(30);Colpodea(10);Colpodea_X(10);Cyrtolophosidida(7);Apocyrtolophosis(7);Apocyrtolophosis_sp.(7);			X	
N18826_2543	Eukaryota(100);Obazoa(81);Opisthokonta(81);Fungi(19);Basidiomycota(5);Agaricomycotina(1);Agaricomycetes(1);Gymnopus(0);Gymnopus_luxurians(0);			X	
N267838_9264	Eukaryota(100);Eukaryota_X(93);Ancyromonadida(93);Ancyromonadida_X(93);Ancyromonadida_XX(93);Ancyromonadida_XXX(93);Ancyromonadida_Group-1(93);Ancyromonadida_Group-1_X(93);Stygamoeba_regulata(93);			X	
N356762_7538	Eukaryota(100);Excavata(97);Discoba(97);Euglenozoa(97);Kinetoplastea(97);Trypanosomatida(64);Trypanosomatidae(64);Blechomonas(37);Blechomonas_campbelli(14);			X	
N350391_18851	Eukaryota(100);TSAR(98);Stramenopiles(98);Gyrista(98);Synchromophyceae(84);Synchromophyceae_X(84);Synchromophyceae_XX(84);Synchromophyceae_XXX(84);Synchromophyceae_XXX_sp.(84);			X	
N14552_33087	Eukaryota(100);Excavata(100);Discoba(100);Euglenozoa(100);Kinetoplastea(100);Trypanosomatida(100);Trypanosomatidae(100);Crithidia(42);Crithidia_brevicula(42);			X	
N66399_9636	Eukaryota(100);Archaeplastida(100);Chlorophyta(100);Chlorophyta_X(100);Chlorophyta_XX(100);Chlorophyta_XXX(100);Chlorophyta_XXXX(100);Scotinosphaera(100);Scotinosphaera_sp.(65);			X	
N40144_5859	Eukaryota(100);Obazoa(97);Opisthokonta(97);Fungi(84);Opisthosporidia(48);Microsporida(48);Microsporida_X(48);Paramicrosporidium(48);Paramicrosporidium_saccamoebae(48);			X	
N89763_77814	Eukaryota(100);TSAR(99);Rhizaria(99);Cercozoa(99);Filosa-Thecofilosea(99);Cryomonadida(99);Rhogostoma-lineage(96);Rhogostoma(93);Rhogostoma_minus(93);			X	
N262889_253	Eukaryota(100);TSAR(98);Stramenopiles(98);Gyrista(98);Chrysophyceae(98);Ochromonadales(85);Ochromonadaceae(76);Urostipulosphaera(50);Urostipulosphaera_granulata(27);			X	

N371134_260707	Eukaryota(100);TSAR(100);Stramenopiles(99);Gyrista(99);Olisthodiscophyceae(26);Olisthodiscales(26);Olisthodiscaceae(26);Olisthodiscus(26);Olisthodiscus_luteus(26);			X	
N319414_185545	Eukaryota(100);TSAR(99);Stramenopiles(98);Gyrista(98);Chrysophyceae(64);Paraphysomonadales(24);Paraphysomonadaceae(23);Paraphysomonas(23);Paraphysomonas_foraminifera(21);			X	
N197041_74963	Eukaryota(100);TSAR(100);Stramenopiles(100);Gyrista(100);Synchromophyceae(96);Synchromophyceae_X(96);Synchromophyceae_XX(96);Synchromophyceae_XXX(96);Synchromophyceae_XXX_sp.(96);			X	
N303332_912	Eukaryota(100);Obazoa(100);Opisthokonta(100);Ichthyosporea(87);Dermocystida(87);Rhynosporidae(87);Rhynosporidae_X(87);Amphibiocystidium(49);Amphibiocystidium_sp.(25);			X	
N27840_394	Eukaryota(100);Archaeplastida(100);Chlorophyta(100);Chlorophyta_X(100);Trebouxiophyceae(95);Chlorellales(92);Chlorellales_X(92);Meyerella(34);Meyerella_sp.(30);			X	
N403064_555	Eukaryota(100);Archaeplastida(100);Chlorophyta(100);Chlorophyta_X(100);Chlorophyceae(100);Chlamydomonadales(99);Chlamydomonadales_X(99);Haematococcus(78);Haematococcus_pluvialis(78);			X	
N103686_5547	Eukaryota(100);Obazoa(89);Opisthokonta(89);Fungi(71);Opisthosporidia(30);Microsporida(30);Microsporida_X(30);Paramicrosporidium(30);Paramicrosporidium_saccamoebae(30);			X	
N322193_6823	Eukaryota(99);Obazoa(91);Opisthokonta(91);Fungi(54);Basidiomycota(37);Agaricomycotina(24);Agaricomycetes(17);Hydnum(0);Hydnum_albomagnum(0);			X	
N271683_1830	Eukaryota(100);TSAR(100);Stramenopiles(100);Gyrista(100);Chrysophyceae(95);Apoikiales(88);Apoikiaceae(88);Apoikiospumella(85);Apoikiospumella_mondseeiensis(80);			X	
N300976_1327	Eukaryota(100);Cryptista(86);Cryptophyta(86);Cryptophyta_X(86);Cryptophyceae(86);Cryptophyceae_X(85);Basal_Cryptophyceae-1(63);Basal_Cryptophyceae-1_X(63);Basal_Cryptophyceae-1_X_sp.(63);			X	
N222328_244634	Eukaryota(100);TSAR(99);Stramenopiles(99);Gyrista(99);Chrysophyceae(97);Chrysophyceae_env_clade-I(91);Chrysophyceae_env_clade-I_X(91);Chrysophyceae_env_clade-I_XX(91);Chrysophyceae_env_clade-I_XX_sp.(91);			X	
N264475_103880	Eukaryota(100);TSAR(97);Stramenopiles(97);Gyrista(96);Chrysophyceae(96);Ochromonadales(71);Ochromonadaceae(62);Poteriospumella(34);Poteriospumella_lacustris(34);			X	
N94821_291	Eukaryota(100);TSAR(80);Stramenopiles(79);Gyrista(79);Phaeothamniophyceae(20);Phaeothamniales(20);Phaeothamniales_X(20);Phaeoschizochlamys(20);Phaeoschizochlamys_santosii(20);			X	
N27500_8046	Eukaryota(100);TSAR(95);Stramenopiles(93);Gyrista(92);Eustigmatophyceae(29);Eustigmatophyceae_X(29);Eustigmatophyceae_XX(29);Pseudotetraedriella(5);Pseudotetraedriella_kamillae(5);			X	
N399473_5405	Eukaryota(100);TSAR(100);Stramenopiles(100);Gyrista(100);Chrysophyceae(98);Chrysophyceae_env_clade-I(83);Chrysophyceae_env_clade-I_X(83);Chrysophyceae_env_clade-I_XX(83);Chrysophyceae_env_clade-I_XX_sp.(83);			X	
N152098_5066	Eukaryota(100);TSAR(87);Stramenopiles(87);Gyrista(85);Phaeothamniophyceae(6);Phaeothamniales(6);Phaeothamniales_X(6);Phaeoschizochlamys(6);Phaeoschizochlamys_santosii(6);			X	
N90909_4968	Eukaryota(100);Cryptista(83);Cryptophyta(83);Cryptophyta_X(83);Cryptophyceae(83);Cryptophyceae_X(82);Basal_Cryptophyceae-1(77);Basal_Cryptophyceae-1_X(77);Basal_Cryptophyceae-1_X_sp.(77);			X	
N120889_2446	Eukaryota(100);TSAR(97);Alveolata(96);Ciliophora(93);Oligohymenophorea(87);Scuticociliatia_1(85);Philasterida(78);Uronemella(73);Uronemella_filificum(73);			X	
N80216_3183	Eukaryota(100);Cryptista(90);Cryptophyta(90);Cryptophyta_X(90);Cryptophyceae(90);Cryptophyceae_X(90);Basal_Cryptophyceae-1(77);Basal_Cryptophyceae-1_X(77);Basal_Cryptophyceae-1_X_sp.(77);			X	
N342892_1668	Eukaryota(100);TSAR(100);Stramenopiles(100);Gyrista(100);Chrysophyceae(97);Paraphysomonadales(94);Paraphysomonadaceae(94);Paraphysomonas(94);Paraphysomonas_mikadiforma(43);			X	
N377106_1357	Eukaryota(100);TSAR(98);Stramenopiles(97);Gyrista(97);Chrysophyceae(91);Paraphysomonadales(82);Paraphysomonadaceae(82);Paraphysomonas(82);Paraphysomonas_mikadiforma(48);			X	
N144678_1111	Eukaryota(100);Cryptista(85);Cryptophyta(85);Cryptophyta_X(85);Cryptophyceae(85);Cryptophyceae_X(85);Basal_Cryptophyceae-1(77);Basal_Cryptophyceae-1_X(77);Basal_Cryptophyceae-1_X_sp.(77);			X	
N406985_873403	Eukaryota(100);TSAR(100);Alveolata(100);Apicomplexa(100);Gregarinomorphea(100);Eugregarinorida(100);Eugregarinorida_EUG3(90);Eugregarinorida_EUG3_X(90);Eugregarinorida_EUG3_X_sp.(90);			X	
N75799_624	Eukaryota(100);TSAR(100);Stramenopiles(100);Gyrista(99);Chrysophyceae(99);Ochromonadales(76);Ochromonadaceae(67);Urostipulosphaera(33);Urostipulosphaera_sp.(15);			X	
N39938_631	Eukaryota(94);Excavata(63);Discoba(62);Euglenozoa(58);Euglenida(58);Petalomonadida(54);Scytomonadidae(54);Scytomonas(20);Scytomonas_saepesedens(20);			X	
N74643_14771	Eukaryota(91);TSAR(70);Stramenopiles(70);Gyrista(69);Pirsoniales(31);Pirsoniales_X(31);Pirsoniales_XX(31);Pirsonia(31);Pirsonia_guinardiae(16);			X	

N284642_6144	Eukaryota(99);TSAR(97);Stramenopiles(97);Gyrista(97);Chrysophyceae(84);Apoikiales(36);Apoikiaceae(36);Apoikiospumella(11);Apoikiospumella_mondseeiensis(11);			X	
N94292_2127	Eukaryota(100);TSAR(100);Stramenopiles(99);Gyrista(99);Coscinodiscophyceae(57);Aulacoseirales(54);Aulacoseiraceae(54);Aulacoseira(54);Aulacoseira_nyassensis(45);			X	
N25953_2992	Eukaryota(100);TSAR(92);Alveolata(92);Ciliophora(77);Oligohymenophorea(42);Oligohymenophorea_X(32);Oligohymenophorea_XX(32);Dexiotricha(32);Dexiotricha_cf._granulosa(32);			X	
N303234_1360	Eukaryota(98);TSAR(77);Stramenopiles(77);Gyrista(77);Pirsoniales(28);Pirsoniales_X(28);Pirsoniales_XX(28);Pirsonia(28);Pirsonia_guinardiae(10);			X	
N418815_1340	Eukaryota(100);TSAR(100);Stramenopiles(100);Gyrista(100);Xanthophyceae(100);Xanthophyceae_X(100);Xanthophyceae_XX(100);Ophiocytium(64);Ophiocytium_parvulum(43);			X	
N88315_1159	Eukaryota(100);TSAR(99);Stramenopiles(99);Gyrista(99);Chrysophyceae(91);Paraphysomonadales(86);Paraphysomonadaceae(86);Paraphysomonas(86);Paraphysomonas_longispina(48);			X	
N97688_1006	Eukaryota(99);Excavata(77);Discoba(76);Euglenozoa(70);Euglenida(70);Euglenida_X(59);Anisonemidae(58);Anisonema(58);Anisonema_sp.(58);			X	
N412035_893	Eukaryota(100);TSAR(98);Stramenopiles(98);Gyrista(98);Chrysophyceae(94);Apoikiales(79);Apoikiaceae(79);Apoikiospumella(61);Apoikiospumella_sp.(51);			X	
N277782_797	Eukaryota(99);TSAR(84);Stramenopiles(84);Gyrista(82);Phaeothamniophyceae(9);Phaeothamniales(9);Phaeothamniales_X(9);Phaeoschizochlamys(8);Phaeoschizochlamys_santosii(8);			X	
N334401_528	Eukaryota(100);TSAR(100);Stramenopiles(100);Gyrista(100);Chrysophyceae(99);Paraphysomonadales(99);Paraphysomonadaceae(99);Paraphysomonas(99);Paraphysomonas_vulgaris(99);			X	
N56833_424	Eukaryota(98);TSAR(91);Stramenopiles(91);Gyrista(89);Phaeothamniophyceae(16);Phaeothamniales(16);Phaeothamniales_X(16);Phaeoschizochlamys(16);Phaeoschizochlamys_santosii(16);			X	
N390932_327	Eukaryota(100);Obazoa(87);Opisthokonta(87);Choanoflagellata(6);Choanoflagellatea(6);Craspedida(6);Monosigidae_Group_A(6);Codosiga(6);Codosiga_hollandica(5);			X	
N172385_311	Eukaryota(100);Obazoa(97);Opisthokonta(97);Fungi(62);Opisthosporidia(18);Microsporida(18);Microsporida_X(18);Paramicrosporidium(17);Paramicrosporidium_saccamoebae(17);			X	
N211745_255	Eukaryota(98);TSAR(76);Alveolata(72);Apicomplexa(11);Coccidiomorphea(8);Adeleida(8);Dactylosomatidae(8);Dactylosoma(8);Dactylosoma_sp.(5);			X	
N179221_403	Eukaryota(98);TSAR(72);Alveolata(47);Dinoflagellata(41);Dinophyceae(41);Peridiniales(15);Amphidomataceae(5);Azadinium(5);Azadinium_cuneatum(3);			X	
N156114_364	Eukaryota(97);TSAR(77);Stramenopiles(73);Bigyra(70);Sagenista(70);Labyrinthulomycetes(70);Amphifilaceae(70);Sorodiplophrys(53);Sorodiplophrys_stercorea(53);			X	
N+A45:A10426273_160	Eukaryota(100);Obazoa(94);Opisthokonta(94);Fungi(46);Opisthosporidia(15);Microsporida(15);Microsporida_X(15);Paramicrosporidium(15);Paramicrosporidium_saccamoebae(15);			X	
