# Supplementary material for: Freshwater and Sediment Host Distinct Yet Overlapping Microeukaryotic Communities, With Sediment Communities Less Impacted by Treated Wastewater
Source: J Eukaryot Microbiol. 2026 Feb 24;73(2):e70070. doi: 10.1111/jeu.70070 (PMC12932744; doi:10.1111/jeu.70070)
Supplement: Supplementary file 13 — Table S4: Showing the organismic groups that were found exclusively in treated wastewater and their relative proportion. [file JEU-73-e70070-s010.pdf]

Group	Relative abundance (%)	Including indicative species (bootstrap >60) for treated wastewater
Gyrista	48.289	*Apoikiospumella mondseeiensis, Nanofrustulum shiloi,* *Paraphysomonas longispina,P. variosa, P. Vulgaris*
Chrysophyceae	36.686	
Euglenozoa	3.317	
Ciliophora	3.062	*Euloptes elegans, Uronemella filificum*
Ascomycota	1.915	
Apicomplexa	1.523	
Fungi	1.176	
Basidiomycota	1.033	*Heterobasison parviporum, Rhogostoma minus,* *Trichosporon cuatenum*
Cryptophyta	0.594	
Amoebozoa	0.472	
Bacillariophyceae	0.367	
Cercozoa	0.363	*Cercomonas braziliensis, Euglypha acanthophora*
Peronosporomycetes	0.271	
Bigyra	0.132	
Discoba	0.125	
Ichthyosporea	0.125	
Dinoflagellata	0.119	
Choanoflagellata	0.113	
Chlorophyta	0.067	*Haematococcus pluvialis*
Chytridiomycota	0.028	
Foraminifera	0.006	
Others	2.999	*Stygamoeba regulata*
